# Gene database for the development of genetic testing for hypertrophic cardiomyopathy

**DOI:** 10.1186/1471-2164-15-S2-P65

**Published:** 2014-04-02

**Authors:** Justin Carlus Silas, Khalid Moghem Al Harbi

**Affiliations:** 1 Centre for Genetics and Inherited Diseases (CGID), Taibah University, Madinah, Kingdom of Saudi Arabia

## Background

Hypertrophic cardiomyopathy (HCM) is a disease characterised by hypertrophy of the left ventricle of the heart. HCM has a prevalence of ~1 in 500 in the general population and is the leading cause of sudden cardiac death (SCD). Mutations in 30 genes have been found to be associated with HCM and accounting for 50-60% genetic causes [1]; etiologies of a larger percentage of HCM still remain unknown. These observations prompted us to create a gene database on genes implicated in HCM and to predict the function of the mutated genes. The findings of more new genes directly or indirectly implicated in HCM will be more helpful in developing comprehensive genetic tests for HCM.

## Materials and methods

We used the search terms “Hypertrophic cardiomyopathy”, “HCM” from the PubMed, Online Mendelian Inheritance in Man (OMIM) [2] and peer-reviewed reports. Functional references from the research articles and the PANTHER [3].

## Results

List of 70 genes reported to be associated with HCM and their function, references, gene network interaction would be available at the time presentation.

## Conclusions

To date most of the research data show only limited number of gene tests are routinely used for HCM and accounting for 50-60% genetic causes. This is because of the lack of a comprehensive gene database available for HCM. Here, for the first time, we present a database that provides an account of 70 currently known genes, which are either directly or indirectly implicated in HCM and it will be helpful in developing comprehensive genetic tests for HCM.
